# Construction of a Highly Stable Water-Based Release Agent via 1:1 Silicone Oil-Cyclotetrasiloxane Synergy

**DOI:** 10.3390/molecules30173509

**Published:** 2025-08-27

**Authors:** Can Wang, Yutong Han, Xiaojuan Du, Sihan Guo, Qiming Zhao, Xiao Chen

**Affiliations:** 1 Microfluidic Synthesis and Separation Laboratory, College of Chemistry and Environment, Southwest Minzu University, Chengdu 610225, China; 18020910720@163.com (C.W.); 19008206834@163.com (Y.H.); t3166205032@163.com (X.D.); hanzai2003@163.com (S.G.); 18782069061@163.com (Q.Z.); 2 Key Laboratory of Basic Chemistry, National Ethnic Affairs Commission, School of Chemistry & Environment, Southwest Minzu University, Chengdu 610225, China

**Keywords:** water-based mold release agent, octamethylcyclotetrasiloxane, polyurethane foaming, film-forming mechanism, stability

## Abstract

This study develops a high-performance water-based mold release agent for polyurethane (PU) foaming applications. We demonstrate that incorporating octamethylcyclotetrasiloxane (D4) into a dimethyl silicone oil emulsion (5 vol% fixed concentration) significantly enhances key performance metrics. By systematically varying D4 content (0–15 vol%), we characterize droplet morphology, particle size distribution, contact angle, and viscosity to elucidate the underlying enhancement mechanism. Our findings reveal the following: (i) Optimal emulsion stability: At 5 vol% D4, the mold release agent exhibits a narrow particle size distribution (6–9 μm). (ii) Efficient processing: Film formation completes within 10 min, reducing demolding force and yielding PU foam with defect-free, non-adherent surfaces. (iii) Storage stability: After 60 days in ambient conditions, performance remains unchanged, with no phase separation observed under thermal stress (60 °C) or refrigeration (2–6 °C). This work explores an alternative pathway to mitigate key limitations—slow film formation and poor shelf-life—offering a prototype for next-generation release agents.

## 1. Introduction

Polyurethane (PU) is a polymer synthesized via condensation polymerization of polyisocyanate and polyol [1,2,3]. Its versatile performance makes it suitable for broad applications across components such as seats and cushioning materials [4,5,6], as well as thermal insulation, coatings, and sealing systems [7]. With accelerating industrialization across sectors, demands for enhanced efficiency and quality in PU molding processes—particularly during demolding—have intensified [8]. We can consider batch molding of soft PU foam as a representative case: reactive components are injected into molds after mixing [9]. Critically, mold surfaces require pre-application of release agents prior to foaming-molding. This practice optimizes production cycles, enhances product surface quality, extends mold service life, and increases manufacturing capacity [10].

According to carrier medium classification, release agents are primarily categorized as solvent-based or water-based systems [11,12]. Traditional formulations predominantly employ solvent-based carriers—exemplified by Nabel A. Negm’s development of cost-effective, low-surface-tension variants with superior emulsification capabilities [13]. However, inherent toxicity and significant environmental impacts are driving the replacement of solvent-based agents by water-based alternatives [12,14]. Despite the environmental advantages and enhanced safety of contemporary water-based systems, critical limitations persist: (i) High aqueous surface tension causes non-uniform coating deposition, compromising demolding efficiency. (ii) Strong intermolecular hydrogen bonding impedes rapid water evaporation during film formation, degrading coating integrity. (iii) Reactive isocyanates form unstable carbamates that decompose into amines and CO_2_, generating product defects including bubbles, shrinkage cavities, and voids [14]. Notably, under increasingly stringent global environmental regulations, water-based agents—characterized by low volatile organic compounds (VOCs) and solvent-free and fluorine-free properties—are continuously replacing solvent-based products [15]. Consequently, developing water-based release agents that combine high demolding performance, exceptional stability, facile preparation, and low cost represents a current research imperative.

Water-based mold release agents fundamentally constitute oil-in-water (O/W) emulsions [16], whose demolding mechanism relies on water evaporation after spray application to molds, ultimately forming functional release films [17]. Decades of emulsion research advancements have critically underpinned mold release agent development: A. Olietti et al. [12] engineered a wax-based waterborne system that mitigated coating inhomogeneity, while Ansar Abbas et al. [18] subsequently reported a wax-free variant exhibiting exceptional room-temperature stability and demolding performance through emulsifier optimization. Despite these innovations, commercial water-based release agents still exhibit persistent shortcomings in demolding efficacy, environmental compatibility, and stability.

To address these limitations, we developed a novel water-based release agent that incorporates, for the first time, a synergistic formulation of dimethyl silicone oil and octamethylcyclotetrasiloxane (D4). The silicone oil provides an inherent film-forming capability, while the strategic addition of D4 enhances both emulsion stability and demolding performance. We systematically varied D4 content ratios and conducted comprehensive characterization—including particle size analysis, contact angle measurements, film formation kinetics, and demolding force quantification—to identify the optimal formulation. Storage stability was evaluated through longitudinal particle size monitoring over 30 days. Furthermore, accelerated stability assessments of the optimal formulation were performed under refrigeration (4 °C) and elevated temperature (60 °C) conditions to evaluate thermal resilience.

## 2. Discussion

### 2.1. The Stability of the Release Agent

#### 2.1.1. Storage Stability

**Figure 1:**
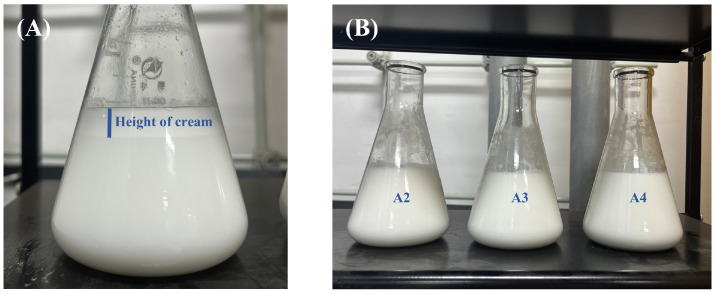
Emulsion stability at 24 h post-preparation: (**A**) A1 (0% D4); (**B**) A2–A4 (5%, 10%, 15% D4, left–right).

**Figure 2:**
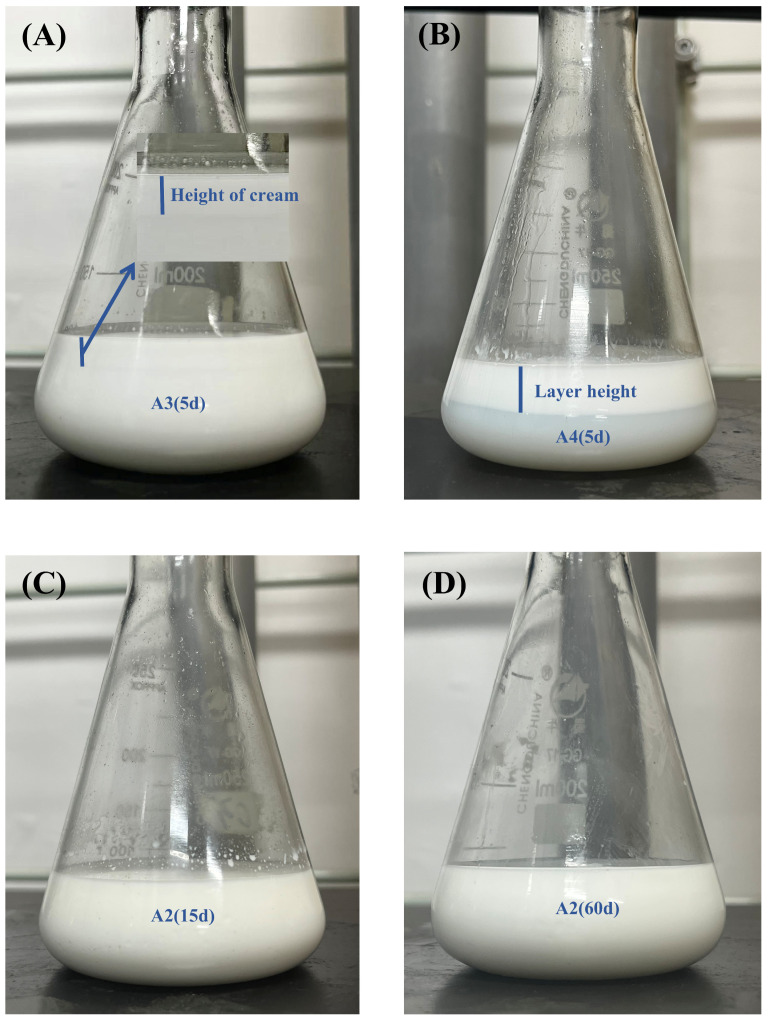
Room-temperature storage stability: (**A**) A3 at 5 days; (**B**) A4 at 5 days; (**C**) A2 at 15 days; (**D**) A2 at 60 days.

To establish the optimal dimethyl–silicone oil ratio, systematic investigations were conducted with its content constrained to ≤15%. Results demonstrated that due to adhesion phenomena observed in polyurethane foam molding at 10% and 15% silicone oil content, the formulations failed to function as release agents. At 5% oil content, the formulation exhibited optimal comprehensive performance and was therefore selected for subsequent analysis. Emulsion stability under ambient storage showed distinct temporal variations: within 24 h, sample A1 displayed obvious precipitation (Figure 1A), while A2–A4 remained visually stable (Figure 1B, left to right: A2/A3/A4). By day 5, A3 developed precipitation (Figure 2A) and A4 underwent complete stratification (Figure 2B). Notably, A2 maintained exceptional stability—no precipitation or stratification was observed through day 15 (Figure 2C) and extending to day 60 (Figure 2D).

#### 2.1.2. Optical Micrograph Analysis

**Figure 3:**
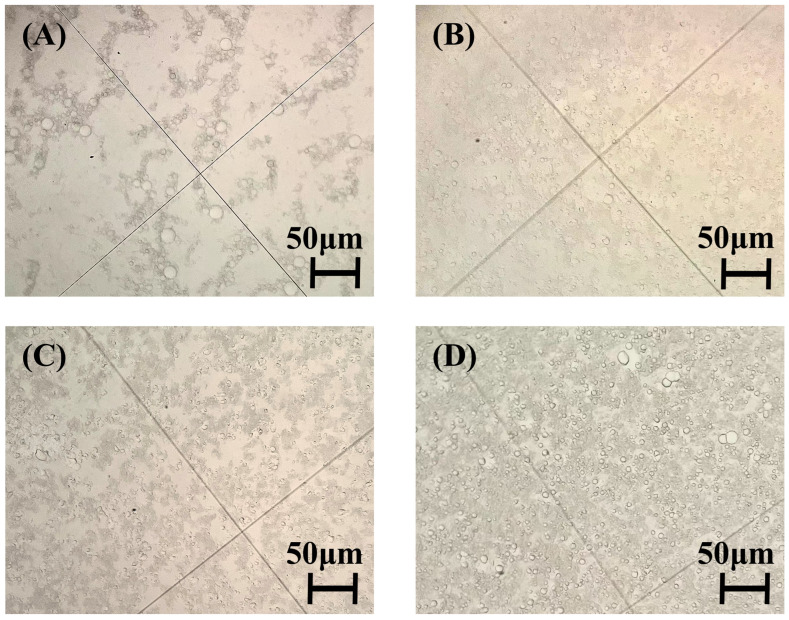
Polarizing microscopy images at 24 h: (**A**) A1 (0% D4); (**B**) A2 (5% D4); (**C**) A3 (10% D4); (**D**) A4 (15% D4).

As shown in Figure 3, all samples exhibited particle size distributions ranging from 6 to 14 μm. The D4-free sample (Figure 3A) displayed significant droplet agglomeration, characterized by small particles adsorbing onto larger ones, resulting in poor dispersibility and non-uniform size distribution. In contrast, samples containing 10% and 15% D4 (Figure 3C,D) showed irregular droplet morphology due to excessive D4 inducing mutual droplet compression and disruption of the original distribution pattern. Notably, the 5% D4 formulation (Figure 3B) demonstrated optimal characteristics: uniformly dispersed spherical particles with a narrow size distribution. This enhancement originates from D4 molecules localizing at particle interfaces, providing spatial steric hindrance that impedes agglomeration kinetics and substantially improves emulsion stability [12,19].

#### 2.1.3. Particle Size Distribution

**Figure 4:**
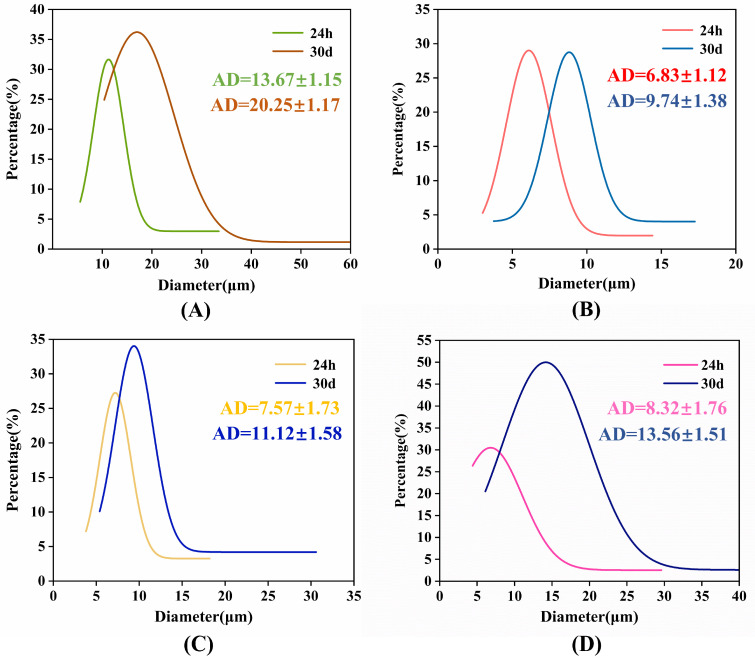
Particle size evolution during ambient storage: (**A**) A1 (0% D4); (**B**) A2 (5% D4); (**C**) A3 (10% D4); (**D**) A4 (15% D4).

To elucidate stability differences and particle size distribution characteristics, Figure 4 presents Nano Measurer-generated distribution maps derived from polarizing microscopy (PM) images at 24 h and 30-day intervals post-preparation. This methodology provides accurate particle size distribution representation. Sample A1 exhibited markedly increased particle sizes and non-uniform distribution (Figure 4A). Conversely, the 5% D4 formulation (A2) demonstrated concentrated particle distribution with smaller diameters (6–9 μm average, Figure 4B), maintaining consistent size ranges throughout storage—indicating sustained small particle dimensions and homogeneity that underpin its higher stability.

Elevated D4 concentrations (10–15%) reduced stability: the 10% D4 sample increased from 7 to 11 μm (Figure 4C), while 15% D4 rose from 8 to 13 μm (Figure 4D) over 30 days. Although both showed improved storage stability versus A1, their distribution uniformity (Figure 3C,D) remained inferior to A2 (Figure 3B). Thus, excessive D4 content (>5%) fails to enhance particle size reduction, distribution homogeneity, or long-term stability.

**Figure 5:**
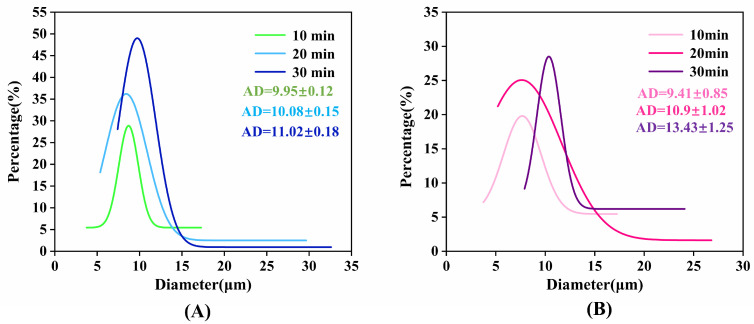
Particle size distribution of A2 under thermal stress: (**A**) 2–6 °C; (**B**) 60 °C.

Figure 5 illustrates the particle size evolution of sample A2 under refrigeration (2–6 °C) and elevated temperature (60 °C) conditions. Refrigerated storage (Figure 5A) induced minimal particle growth (from 10 to 11 μm), while controlled size increase occurred at 60 °C (from 9 to 13 μm). Statistical analysis via one-way ANOVA revealed no significant differences in particle size across 10, 20, and 30 min intervals at both 2–6 °C and 60 °C (Table 1). Collectively, these constrained particle size variations under extreme temperatures indicate limited droplet coalescence, preserving monodispersity. This thermal tolerance expands the operational range, suggesting a potential pathway to address key limitations.

### 2.2. Contact Angle and Viscosity Analysis

**Figure 6:**
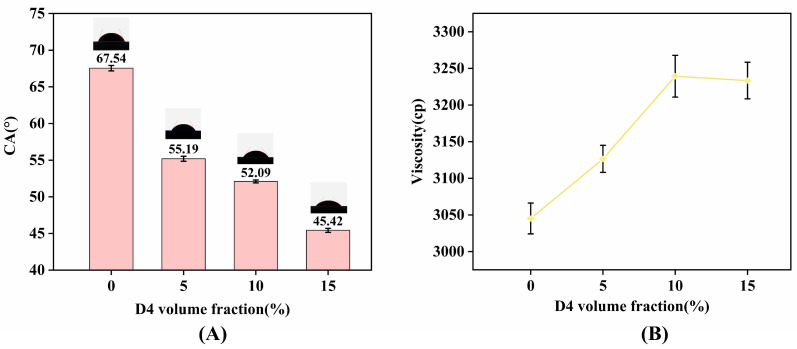
Contact angle (CA) and viscosity versus D4 volume fraction: (**A**) CA on aluminum substrates; (**B**) emulsion viscosity.

Figure 6A presents instantaneous contact angles for all samples. Consistent with characteristics of O/W emulsions, contact angle magnitude correlates with wetting properties—a parameter directly linked to enhanced demolding efficacy and stability [19,20]. The D4-free sample exhibited the maximal contact angle (θ_max_), while the 15% D4 formulation yielded the minimal angle (θ_min_). According to Young’s equation, surface tension (γₗᵥ) shows positive correlation with θ. This confirms D4 effectively reduces γₗᵥ, yet optimal concentration control remains critical: excessively low θ (indicating ultra-low γₗᵥ) promotes non-uniform spreading on mold surfaces. Moreover, diminished contact angles reduce the energy barrier for particle detachment from interfaces, explaining the compromised stability of A3 and A4 [21].

Figure 6B demonstrates a positive correlation between emulsion viscosity and D4 content. While elevated viscosity inhibits droplet coalescence and sedimentation [22], excessive values degrade stability. This viscosity–stability relationship aligns with observations from Figure 1 and Figure 2: the D4-free sample (low viscosity) sedimented within 24 h (Figure 1A); A4 (15% D4, highest viscosity) stratified by day 5 (Figure 2B); and A3 (10% D4) exhibited minor sedimentation (Figure 2A) and significant particle growth after 30 days (Figure 4C). This phenomenon arises because high viscosity impedes droplet fragmentation during homogenization, resulting in larger initial droplet sizes that accelerate emulsion stratification and sedimentation [23]. Crucially, the 5% D4 formulation (A2) maintains optimal viscosity, balancing stability preservation with demolding efficacy.

### 2.3. Surface Tension Measurement

**Figure 7:**
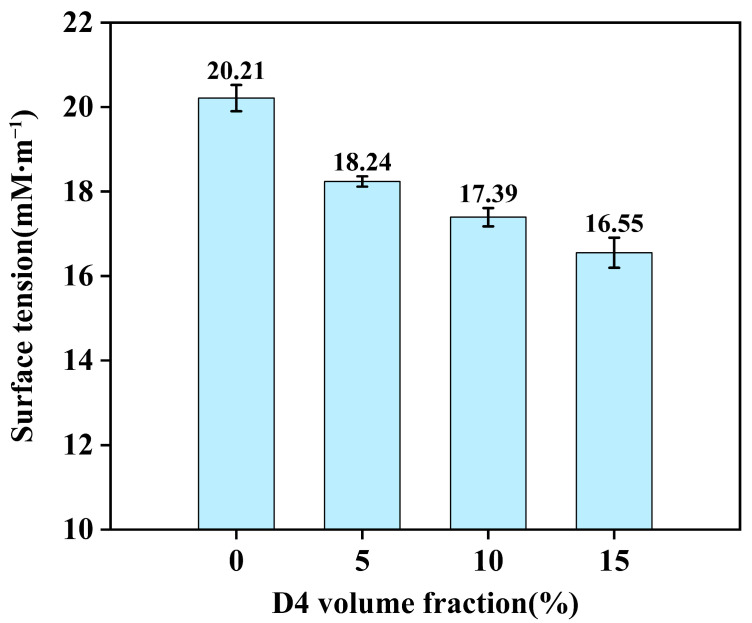
Surface tension variation in oil-phase versus D4 content.

To verify the distribution characteristics of D4 at phase interfaces, surface tension measurements were conducted on the oil phases of different samples (Figure 7). Results indicate that D4 introduction induced a modest reduction in oil-phase surface tension. Further application of Antonov’s rule (Equation (1)) revealed no significant differences in calculated interfacial tension values derived from water–oil phase surface tensions. Collectively, these findings suggest that D4 primarily localizes at phase interfaces, yet its core mechanism does not operate through substantial interfacial tension reduction.

(1) $$\gamma_{o,w}=\left|\gamma_{o}-\gamma_{w}\right|\;$$

### 2.4. Demoulding Behavior

**Figure 8:**
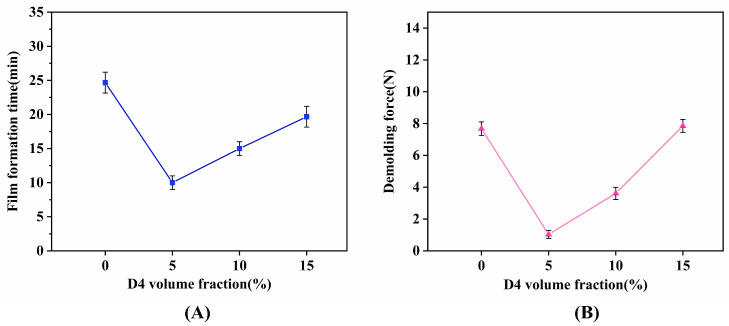
Processing parameters versus D4 volume fraction: (**A**) film formation time; (**B**) demolding force.

Figure 8A reveals a nonlinear relationship between D4 content and film-forming time. Despite reduced contact angles at 10–15% D4 (Figure 6A), excessive D4 disrupts dimethyl silicone oil distribution—inducing silicone oil coalescence and extending film formation after spraying. Conversely, A2 exhibits minimal film-forming time and optimal demolding performance. This demonstrates that controlled D4 incorporation (5%) accelerates aqueous phase evaporation, shortens film formation kinetics, and directly improves production efficiency.

Figure 8B illustrates demolding force variation across D4 concentrations. Demolding forces were substantially elevated at 0% and 15% D4; conversely, the 5% formulation required merely 1 N, facilitating the effortless removal of cured polyurethane foam.

**Figure 9:**
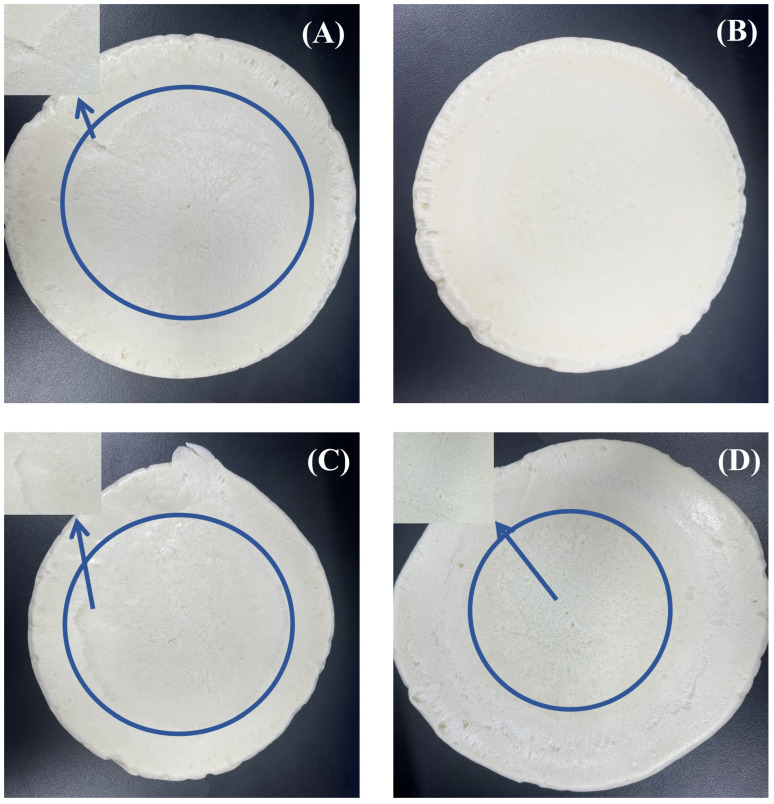
Demolded product surface morphology: (**A**) A1 (0% D4); (**B**) A2 (5% D4); (**C**) A3 (10% D4); (**D**) A4 (15% D4).

Figure 9A reveals severe mold adhesion and discontinuous film formation with the A1 release agent. While A3/A4 formulations reduced adhesion area (Figure 9C,D), they introduced excessive surface porosity in polyurethane products. Mechanistically, low-molecular-weight D4 penetrates silicone oil molecular chains, disrupting ordered packing and compromising film continuity. At elevated concentrations (>5%), this creates defective films with non-uniform pore distribution that degrade demolding performance.

Crucially, the 5% D4 formulation (A2) achieves optimal performance: minimal film-forming time (Figure 8A), the lowest release force (1N, Figure 8B), and pore-free surfaces (Figure 9B). The integrated contact angle and viscosity data (Figure 6) empirically demonstrate that 5% D4 precisely optimizes the wettability–spreadability balance, enabling uniform mold surface coverage. This resolves the inherent limitation of water-based systems where high aqueous surface tension compromises mold wetting.

**Figure 10:**
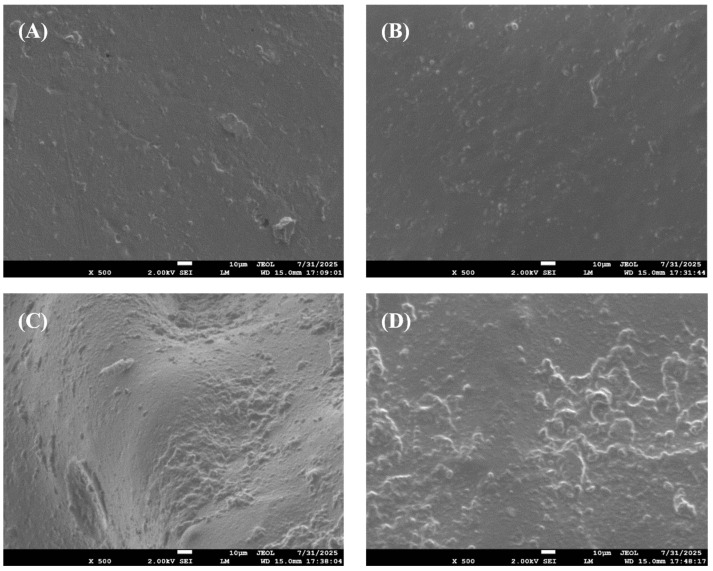
SEM images of polyurethane foam surfaces: (**A**) A1 (0% D4); (**B**) A2 (5% D4); (**C**) A3 (10% D4); (**D**) A4 (15% D4).

To microscopically evaluate film formation quality, SEM imaging was performed on polyurethane foam surfaces, analyzing film structure, continuity, and smoothness (Figure 10). Figure 10C exhibits pronounced depressions with elevated surface roughness. Figure 10D shows no major depressions but contains numerous cavities. Figure 10A demonstrates relatively improved yet still suboptimal morphology with minor pits. Critically, Figure 10B reveals notably smoother surfaces at 5% D4 content, where only minimal microscopic voids were observed at 500× magnification. These observations indicate that continuous and smooth PDMS films formed at 5% D4 content facilitate complete demolding, whereas other formulations induce surface defects including cavities and bubbles. Additionally, the absence of significant bubble formation in A2 suggests limited chemical reaction between active components and isocyanates during foaming—extensive reactions would theoretically generate substantial gas evolution and severe demolding complications.

## 3. Materials and Methods

### 3.1. Materials and Instruments

The water-based mold release agent was formulated using dimethyl silicone oil (AR grade), Span 80 (99%), Tween 80 (AR grade), octamethylcyclotetrasiloxane (D4, 98%) from Shanghai Titan Technology (Shanghai, China), and deionized water. Experimental instrumentation comprised an electronic balance (AE240, Shanghai Jingtian, Shanghai, China); high-speed dispersion homogenizer (FJ200-SH, Shanghai Specimen and Model Factory, Shanghai, China); custom polyurethane foaming machine; polarizing microscope (XPR500C, Shanghai Caikang Optical, Shanghai, China); digital viscometer (NDJ-8S, Shanghai Jingtian, Shanghai, China); digital force gauge (VICTOR10N, Shenzhen Yisheng Shengli, Shenzhen, China); dynamic contact angle analyzer (TY-C11, Shandong Tianyan, Zhucheng, China); automatic surface tensiometer (BZY-1, Shanghai Hengping Instrument, Shanghai, China); and field-emission scanning electron microscope (SU9000, Hitachi, Tokyo, Japan).

### 3.2. Preparation of the Release Agent

The formulation of the novel water-based mold release agent developed in this study is detailed in Table 2. Preparation proceeded as follows:

(i) Solution preparation: Dimethyl silicone oil (5 mL), Span-80 (5 mL), Tween-80 (2 mL), and octamethylcyclotetrasiloxane (D4, 0–15 mL) were metered. Deionized water was added to adjust the final volume to 100 mL.
(ii) Emulsification: All components were transferred to a 250 mL conical flask and subjected to homogenization at 2000 rpm for 2 min at room temperature, yielding a homogeneous emulsion.

### 3.3. Sample Characterization

#### 3.3.1. Polarizing Microscope Observation

The sample surface morphology and microstructure were characterized using a polarizing microscope. The procedure involved applying 1–2 drops of emulsion onto a clean glass slide via disposable pipette, carefully lowering a coverslip to minimize air bubble entrapment, and imaging under 100× total magnification (10× eyepiece/10× objective) to document emulsion particle morphology and size distribution.

#### 3.3.2. Testing of Stability

Emulsion stability was systematically evaluated through static storage at ambient temperature (25 ± 2 °C). The time to phase separation (onset of precipitation/stratification) was recorded; a prolonged duration indicates enhanced long-term storage stability and phase separation resistance. For quantitative analysis, emulsion aliquots were examined via polarizing microscopy after 24 h and 30-day storage intervals. Particle size distribution diagrams were generated using Nano Measurer software (v1.2), with minimal temporal variation in distributions indicating optimal physical stability. Temperature resilience was assessed through accelerated testing:

(1) High-temperature stability: Samples were incubated at 60 °C [19] for 10, 20, and 30 min intervals (selected to simulate practical demolding cycles), cooled to ambient temperature, and immediately analyzed for particle size distribution changes.
(2) Refrigeration stability: Parallel evaluation at 2–6 °C [24] following identical time intervals with subsequent particle size analysis.

#### 3.3.3. Contact Angle Measurements

Wetting performance of release agents on foaming mold surfaces was evaluated via contact angle measurements. According to fundamental wetting principles, contact angles <90° indicate hydrophilic surfaces, while >90° signify hydrophobicity. In emulsion systems, angles exceeding 90° facilitate water-in-oil (W/O) emulsions, whereas sub-90° angles promote oil-in-water (O/W) formation—consistent with the O/W emulsion developed in this study. Consequently, reduced contact angles correlate with enhanced wettability and superior system stability in such formulations.

Instantaneous contact angles of the release agent were determined using a dynamic contact angle analyzer, recording values immediately upon emulsion droplet contact with the solid surface. To simulate actual mold conditions and minimize measurement error, aluminum plates matching the mold material served as substrates. Precise 5 μL emulsion aliquots were deposited via a blunt-tipped microsyringe, minimizing gravitational effects on contact angle measurements. Replicate measurements (n = 3) were performed for each sample, with mean values reported as representative data.

#### 3.3.4. Viscosity Measurements

Viscosity represents a critical physical parameter governing emulsion performance. Optimal viscosity suppresses droplet coalescence and sedimentation while preserving emulsion homogeneity. Conversely, elevated viscosity markedly reduces fluidity, inducing application difficulties during spraying and potentially disrupting emulsifier distribution, compromising emulsion stability.

Sample viscosity was quantified using an NDJ-8S digital viscometer (Shanghai Jingtian, Shanghai, China) with rotor #4 selected at 30 r/min. The measurement protocol comprised the following steps:

(i) Transferring 200 mL of sample to a dedicated measurement vessel;
(ii) Engaging rotor #4 at 30 r/min;
(iii) Recording stabilized viscosity readings.

Replicate measurements (n = 3) were performed for each sample, with mean values reported as representative data.

#### 3.3.5. Surface Tension Measurement

Surface tension measurements were conducted using an automatic tensiometer (BZY-1, Shanghai Hengping Instrument) with the platinum plate method. Prior to each measurement, the platinum plate was

(i) Rinsed at a 45° downward angle with deionized water;
(ii) Flame-sterilized until red-hot using an alcohol burner;
(iii) Cooled for 30 s before mounting.

The instrument was calibrated with 30 mL ultrapure water under temperature-controlled conditions (25.0 ± 0.1 °C). Automatic stage elevation was initiated until plate–liquid contact, with calibration validated at 71.90 mN·m^−1^. Following calibration, oil-phase samples (dimethyl silicone oil/D4 mixtures) from four formulations were analyzed. For each sample, a 30 mL aliquot was tested; triplicate measurements were performed (n = 3); and the mean value was calculated as representative data.

A rigorous plate-cleaning protocol was reapplied between tests to minimize cross-contamination.

### 3.4. Demolding Behavior Test

**Figure 11:**
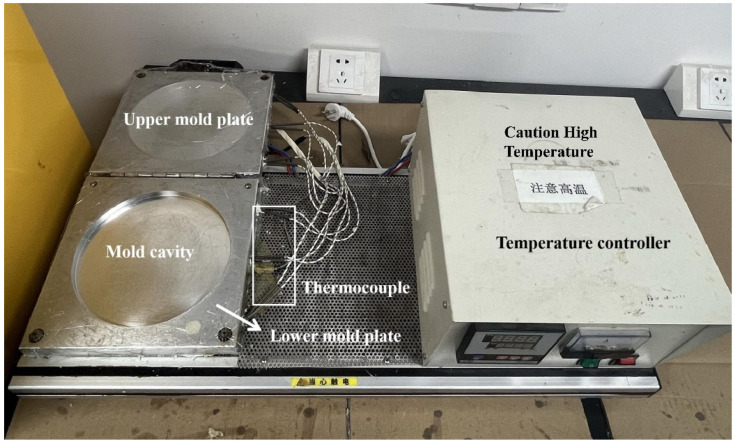
Schematic of customized polyurethane foaming equipment.

Demolding performance was evaluated through three key metrics: film formation time, release force, and demolded product surface quality. The customized polyurethane foaming equipment utilized (Figure 11) was tested via this protocol:

(i) Coating and foaming: Uniformly apply release agent to mold interior. We employed slow-rebound polyurethane with Component A/B ratio of 3:1. After high-speed mixing of polyurethane components A/B to ensure complete homogeneity, rapidly inject mixture into mold cavity. The mold temperature was maintained at 25 °C, yielding a foam density of 75 kg/m^3^.
(ii) Film formation time: Record time required for complete release agent film formation post-curing. Triplicate measurements per sample group were conducted, with mean values reported. Shorter durations indicate higher demolding efficiency.
(iii) Release force measurement: Use a digital force gauge to measure the minimum separation force between the cured foam and the mold. Before the measurement, all the foams underwent a standardized 25 min foaming process to form circular sample shapes. The force gauge fixture was fixed at the center of the foam, and force was applied vertically (90°) at a controlled average speed of 100 ± 10 mm/min. The lower the value, the better the separation performance. Each sample was measured three times, and the average value was calculated.
(iv) Surface quality assessment: Visually inspect demolded products. Optimal performance presents: defect-free surfaces, zero mold residue adhesion, and absence of surface pores/imperfections.

Subsequently, a section of the foamed polyurethane was excised for analysis using field-emission scanning electron microscopy (SEM) to examine film surface morphology and thickness. SEM imaging was performed at an acceleration voltage of 2.0 kV.

## 4. Conclusions

This study developed a novel water-based release agent via a facile synthesis route. The formulation employs dimethyl silicone oil as the primary film-forming agent, octamethylcyclotetrasiloxane (D4) as a synergistic co-additive providing spatial steric hindrance to reduce droplet agglomeration, and Span-80/Tween-80 as compound emulsifiers facilitating stable emulsion formation between oil and aqueous phases. The optimized formulation demonstrates enhanced stability and release performance. Compared to other water-based release agents applicable to polyurethane foaming [24], this agent exhibits lower release forces and superior thermal resilience.

Crucially, a 1:1 volume ratio of D4 to dimethyl silicone oil maximizes comprehensive functionality, yielding these key findings:

(i) D4 incorporation enhances colloidal stability by improving silicone oil particle dispersion and suppressing coalescence kinetics, significantly extending storage and operational stability.
(ii) D4 reduces contact angles and interfacial tension, substantially enhancing mold surface wettability.
(iii) Optimal D4 loading accelerates film formation kinetics and minimizes release forces (1N), boosting production efficiency.

This work demonstrates the potential to address thermal instability and wettability limitations in laboratory-scale tests, laying a foundation for future industrial development.

## Figures and Tables

**Table 1:** Statistical analysis of particle size.

Condition	Temperature	*p*-Value	MaxΔ (μm)	Significance
Refrigeration	2–6 °C	0.135	1.07	No
Heat stress	60 °C	0.122	4.02	No

**Table 2:** Formulation of water-based release agent.

Sample	PDMS (mL)	Span-80 (mL)	Twen-80 (mL)	D4 (mL)	Deionized Water (mL)
A1	5	5	2	0	88
A2	5	5	2	5	83
A3	5	5	2	10	78
A4	5	5	2	15	73

## Data Availability

Data are contained within the article.

## Abbreviations

The following abbreviations are used in this manuscript:

PU	Polyurethane
D4	Octamethylcyclotetrasiloxane
PDMS	Polydimethylsiloxane
CA	Contact Angle
